# A Cautionary Tale: Quantitative LC-HRMS Analytical Procedures for the Analysis of *N*-Nitrosodimethylamine in Metformin

**DOI:** 10.1208/s12248-020-00473-w

**Published:** 2020-07-01

**Authors:** Jingyue Yang, Tim Andres Marzan, Wei Ye, Cynthia D. Sommers, Jason D. Rodriguez, David A. Keire

**Affiliations:** Division of Pharmaceutical Analysis, Food and Drug Administration, 645 S. Newstead Ave., St. Louis, Missouri 63110 USA

**Keywords:** High-resolution mass spectrometry, Nitrosamines, NDMA, Pharmacueticals

## Abstract

A private testing laboratory reported in a Citizen Petition (CP) to FDA that 16 of 38 metformin drug products they tested had *N*-nitrosodimethyl amine (NDMA) amounts above the allowable intake (AI) of 96 ng/day. Because the FDA had been monitoring drugs for nitrosamines, orthogonal analytical procedures had been developed, validated and applied to detect the following nitrosamines in metformin drug products (if present): (i) NDMA (with a dedicated method) or (ii) NDMA (with a second confirmatory method), *N*-nitroso-diethylamine (NDEA), *N*-ethyl-*N*-nitroso-2-propanamine (NEIPA), *N*-nitroso-diisopropylamine (NDIPA), *N*-nitroso-di-n-propylamine (NDPA), *N*-nitroso-methylphenylamine (NMPA), *N*-nitroso-di-n-butylamine (NDBA) and *N*-nitroso-*N*-methyl-4-aminobutyric acid (NMBA). In contrast to the private laboratory results, FDA testing on the same set of 38 samples with orthogonal procedures observed amounts over the AI in only 8 of the 38 products and generally observed lower values than reported by the private testing laboratory. As described here, the investigation into the cause of the discrepancy revealed that *N,N*-dimethylformamide (DMF) can interfere with NDMA measurements. The data showed that the use of sufficient mass accuracy in the data acquisition and appropriate mass tolerance setting in the data processing to assure the selectivity of mass spectrometry measurements of NDMA in the presence of co-eluting DMF was necessary to prevent overestimation of the level of NDMA in metformin drug products. Overall, care should be taken to assure the necessary specificity in analytical procedures for adequate assessment of the nitrosamine level in drug products that also contain DMF or other potential interfering substances.

**Electronic supplementary material:**

The online version of this article (10.1208/s12248-020-00473-w) contains supplementary material, which is available to authorized users.

## INTRODUCTION

NDMA and other nitrosamines are common contaminants in low amounts in foods, beverages, cosmetics, water, tobacco products, and consumer goods (1–4). In 2018, observations of NDMA and NDEA in angiotensin receptor blocker drugs (ARBs) led to recalls of batches of products which had unacceptable amounts of nitrosamines (5,6). Since the ARB nitrosamine impurities were discovered, there have been additional drugs found to contain nitrosamines in the parts-per-million (ppm or ng/mg) to parts-per-billion (ppb or pg/mg) range (e.g., ranitidine in 2019, metformin in 2020 (7)) each with unique properties in terms of the route and source of their presence.

Key to detection of nitrosamines in each drug is the application of appropriate measurement technology focused on detecting low nanogram amounts of nitrosamines in solvents, intermediates, APIs and finished dosage forms. Regulatory agencies and pharmaceutical manufacturing firms around the world have developed and validated analytical techniques focused on nitrosamine detection. Many of the methods developed by pharmaceutical regulatory labs have been made publicly available to speed the risk-based screening of manufacturing processes for nitrosamines (FDA.gov and EU OMCL lab websites (8–11)).

When the FDA became aware of reports from international regulators of NDMA contamination in metformin, the FDA developed, validated, and made publicly available a liquid chromatography high-resolution mass spectrometry (LC-HRMS based on an orbitrap mass analyzer) method that was specific for the detection and quantitation of NDMA in metformin. Additional methods were developed that could detect eight different nitrosamines for confirmatory measurements. To date, only NDMA has been detected in certain metformin products (12).

Most recently, the FDA became aware of reports of higher levels of NDMA in metformin drug products by reports in a Citizen Petition (CP) filed by a private laboratory which reported 16 of 38 metformin drug products tested had NDMA amounts over the allowable intake (see Table 1 last column) using one method (13). The FDA obtained the 38 metformin lots from the private laboratory to confirm their observations with three orthogonal methods (only two of the three FDA methods are reported here). The FDA testing confirmed NDMA levels above the AI in 8 of the 38 lots tested. However, the FDA testing detected below AI or no detectable amounts of NDMA in lots that the private laboratory reported values above the AI. Overall, the FDA observed that the levels of NDMA, when present, were generally lower than those reported by the private laboratory (14). To assess the cause for the discrepancy between the NDMA values measured by the Agency and by the private laboratory, the FDA reproduced the analytical procedure reported by the private laboratory as closely as practical with the available level of detail (15).

**Table 1:** NDMA Amounts in metformin samples reported by FDA (using FDA-1 and FDA-2 methods) and the private laboratory

Sample #	Metformin dosage and formulation	Manufacturer name as per private laboratory	Lot #	FDA-1^a,b^ (ng/mg)	FDA-2 (ng/mg)	Private lab (ng/mg)
1	500 mg IR	ACI Healthcare USA, Inc.	D105061	ND^c^	ND	0.062
2	500 mg IR	ACI Healthcare USA, Inc.	C105019A	ND	ND	ND
3	500 mg IR	ACI Healthcare USA, Inc.	D105019	ND	ND	ND
4	500 mg ER	Actavis Pharma, Inc.	1376339 M	0.021^d^	0.021	0.364
5	750 mg ER	Actavis Pharma, Inc.	1354471A	0.050	0.047	0.427
6	500 mg ER	Aiping Pharmaceutical, Inc.	190300211	ND	ND	ND
7	1000 mg ER	Aiping Pharmaceutical, Inc.	190200411	ND	0.008 ^d^	ND
8	1000 mg IR	Zydus	184759	ND	0.006 ^d^	ND
9	750 mg ER	Amneal Pharmaceuticals, LLC	AM180770A	0.079	0.076	0.600
10	500 mg ER	Amneal Pharmaceuticals, LLC	AM190107AA	0.314	0.292	0.790
11	500 mg ER	Amneal Pharmaceuticals, LLC	HD03319A	0.293	0.255	0.566
12	500 mg ER	Amneal Pharmaceuticals, LLC	HM02918A	0.289	0.265	0.564
13	850 mg IR	Amneal Pharmaceuticals, LLC	AM180405A	ND	ND	0.276
14	500 mg ER	Apotex Corp.	NE5801	0.121	0.112	0.180
15	750 mg ER	Apotex Corp.	NG2595	ND	ND	ND
16	1000 mg IR	Ascend Lab., LLC	4200061B	ND	ND	0.529
17	500 mg IR	Ascend Lab., LLC	4980028B	ND	ND	ND
18	1000 mg IR	Ascend Lab., LLC	4200024C	ND	ND	ND
19	500 mg IR	Aurobindo Pharma	MTSA19016-B	ND	ND	0.060
20	500 mg IR	Aurobindo Pharma	MTSA19070-C	ND	ND	ND
21	500 mg ER	Epic Pharma LLC	190101111	0.010 ^d^	0.008 ^d^	ND
22	500 mg ER	Granules Pharma Inc	4910134A	ND	ND	0.082
23	850 mg IR	Heritage	4510157A	ND	ND	0.299
24	500 mg IR	Heritage	4500753A	ND	ND	0.412
25	1000 mg IR	Heritage	4521630A	ND	ND	ND
26	500 mg ER	Ingenus Pharmaceuticals	19388005	0.012 ^d^	0.009 ^d^	ND
27	500 mg ER	Lupin Pharma	G901203	0.170	0.138	0.244
28	1000 mg IR	Megalith Pharmaceuticals	442180318	ND	ND	ND
29	1000 mg ER	Mylan Pharmaceuticals	3090719	0.011 ^d^	0.010	ND
30	1000 mg ER	Nostrum Labs Inc	MEF290206	ND	ND	ND
31	500 mg ER	Oceanside	19D125P	0.010 ^d^	0.005 ^d^	ND
32	750 mg ER	Sun Pharmaceutical Ind	JKU0880A	ND	ND	ND
33	500 mg ER	Sun Pharmaceutical Ind	JKU2539A	ND	ND	ND
34	500 mg ER	Tagi Pharma Inc	5841,910035	ND	ND	ND
35	500 mg ER	Tagi Pharma Inc	5841905129	0.015 ^d^	0.012	ND
36	500 mg ER	Time Cap Laboratories	XP9004	0.082	0.071	0.106
37	500 mg IR	Westminster Pharmaceuticals	B105067B	ND	ND	ND
38	1000 mg IR	Westminster Pharmaceuticals	B107261B	ND	ND	ND

IR indicates immediate release and ER indicates extended release

^abcd^AI for IR product is 0.038 ng/mg based on an maximum daily dose of 2550 mg; AI for ER product is 0.048 ng/mg based on an maximum daily dose of 2000 mg; ND = Not Detected (< LOD); value ≥ LOD but < LOQ; LOD and LOQ are 0.010 ng/mg and 0.030 ng/mg, respectively for FDA-1, and 0.005 ng/mg and 0.010 μg/g for FDA-2.

## METHODS AND MATERIALS

NDMA standard solution (100 μg/mL in methanol) was purchased from Chem Service (West Chester, PA). Stable isotope labeled NDMA (^13^C_2_; D_6_) (1 mg/mL in CD_2_Cl_2_) was ordered from Cambridge Isotope Laboratories (Andover, MA). *N,N*-Dimethylformamide (DMF) (99.8%) was purchased from Acros Organics, part of Thermo Fisher Scientific (Waltham, MA). Methanol (LC-MS grade) and formic acid (LC-MS grade) were purchased from Fisher Scientific (Hampton, NH). Water (ultrapure, resistivity ≥ 18.2 MΩ.cm) was from an in-house water purification system (ELGA) (Celle, Germany). All the metformin samples tested were commercially available finished drug products (FDP) and are listed in Table 1.

### FDA LC-MS Methods

The full methods used by the FDA to analyze the samples are given in the supporting information section (Supplemental Data_2_methods) so only a brief overview of each method is given here. The primary test—FDA-1 (8)—is a publicly-available liquid chromatography high–resolution mass spectrometry (LC-HRMS) method to determine the level of NDMA in metformin drug products—both immediate and extended release formulations. The alternative method—FDA-2 (8)—is also a publicly available LC-HRMS method that can serve as an orthogonal technique to FDA-1. FDA-2 is designed to screen for eight nitrosamines and uses different chromatographic conditions (column chemistry, gradients, and flow rate). The chromatographic conditions for the two methods are summarized in Table S1 of the supplemental methods document.

For FDA-1 and FDA-2, the same approach was applied for sample preparation, mass spectrometric detection, and quantitation. Samples were prepared by methanol extraction at a ratio of 100 mg API per mL of methanol. Mass spectrometric analyses were performed on a Q Exactive Hybrid Quadrupole-Orbitrap mass spectrometer (Thermo Scientific (Waltham, MA)). The analytes were ionized by electrospray ionization (ESI), and NDMA was detected by parallel reaction monitoring (PRM) scan at a normalized collision energy of 80 and a mass resolution of 35,000. The extracted ion chromatograms (EIC) of NDMA ion at *m/z* 75.0553 with a mass tolerance of ± 15 ppm were used for quantitation by a single external NDMA standard. For the details see supplemental information. For the list of samples tested see Table 1 in the Results section.

### Evaluation of the Private Laboratory LC-MS Method: Preparation of NDMA Calibration Standard Solutions

NDMA calibration standard solutions were prepared as described (15). NDMA and NDMA (^13^C_2_;D_6_) standard solutions were diluted in methanol to prepare nine calibration standard solutions each containing 40 ng/mL NDMA (^13^C_2_;D_6_) and with increasing NDMA concentration levels of 0.3, 0.5, 1, 3, 5, 20, 50, 100, and 200 ng/mL.

### Evaluation of the Private Laboratory LC-MS Method: Preparation of Sample Solutions with Isotopically Labeled Standards

One tablet was crushed for each sample and methanol was added at a ratio of 1 mL methanol per each 100 mg of API. NDMA (^13^C_2_; D_6_) was spiked at a final concentration of 40 ng/mL. The solution was vortexed, shaken with a wrist action shaker for 40 min, and centrifuged at 4500 rpm for 15 min. The supernatant was then transferred to an HPLC vial for analysis. A spiked sample was also prepared by following the procedure above with NDMA spiked at a concentration of 20 ng/mL.

### Replication of the Private Laboratory’s LC-MS Method

The LC-MS method used by the private laboratory for NDMA determination was mimicked in this study. All experiments were performed on a Vanquish Horizon UHPLC system coupled to an Orbitrap Exploris™ 480 mass spectrometer, both manufactured by Thermo Scientific (Waltham, MA). The injection volume was 10 μL and the LC separation was performed on a C18 column (Luna Omega PS C18, 3 μm, 4.6 × 100 mm, Phenomenex (Torrance, CA)). A gradient constituting of water and methanol (both containing 0.1% formic acid) as mobile phase A and B was applied as follows: 0 to 2 min, 2.5% B; 7 min, 50% B; 7.01 min, 50% B; 12 min, 97.5% B; 12.9 min, 97.5% B; 13 min, 2.5% B; and 15 min, 2.5% B. The flow rate was 0.3 mL/min for the first 7 min, changed to 0.75 mL/min at 7.01 min, and stayed at this value for the remaining run time. The column was kept at 40 °C, and the autosampler was set at 5 °C. The analyte was ionized by atmospheric pressure chemical ionization (APCI) in positive ion mode under the following conditions: sheath gas flow rate, 50 arbitrary units; aux gas flow rate, 10 arbitrary units; sweep gas flow, 0; S-lens, 30; current, 4 μA, ion transfer tube temperature, 275 °C; and vaporizer temperature, 400 °C. The ions with m/z value of 75 ± 2 and 83 ± 2 were fragmented with a normalized collision energy of 80, and the mass spectra were acquired in a m/z range of 40–90 at a mass resolution of 45,000.

### Evaluation of the Private Laboratory LC-MS Method: Data Processing and Quantitation

The mass spectrometric data were processed by Xcalibur software (Thermo Scientific, Waltham, MA). The ratio of the extracted ion chromatogram peaks (EIC) of the monoisotopic ions of NDMA (m/z 75.0553) and NDMA (^13^C_2_;D_6_) (m/z 83.0997) was used for quantitation. A mass tolerance window of ± 15 ppm or ± 30 ppm was applied to obtain the EICs. A weighted (1/×) calibration curve was constructed using nine calibration standard solutions and was used to determine the levels of NDMA in samples.

## RESULTS

For the analysis of the 38 lots in this study, the FDA compared values obtained from the primary metformin testing method for NDMA (denoted as FDA-1) and the secondary confirmatory method (FDA-2) which was used to confirm the observations from the primary test. The performance characteristic values reported for FDA-1 and FDA-2 analytical procedures were derived from validation experiments and were shown to be fit-for-the-intended purpose of detecting nitrosamines in metformin products with LOQs at or better than 0.03 ppm. By contrast, the procedure and performance characteristics for the private laboratory method were derived from the information they provided in the Citizen Petition and was only partially verified in this work.

If there was a difference in the amounts of NDMA observed by the primary and secondary FDA analytical procedures for the same sample, then a matrix effect would have been indicated, and more study required. If the results of the primary (FDA-1) and secondary (FDA-2) measurements were similar, the results of the primary screen were considered reportable results. The results from the two independent FDA tests on NDMA amounts in the 38 samples were consistent (Table 1).

By contrast, where NDMA amounts were detected in samples by the private laboratory and by the FDA testing, the NDMA content measurements were consistently higher in the private laboratory results (average of 5-fold greater with a range of 1.3 to 17 times greater for individual samples). In some cases, NDMA was not detected by FDA, but was reported to be present at amounts above the AI by the private laboratory (i.e.*,* Sample #s 1, 13, 16, 19, and 22–24). In other examples, NDMA amounts near the LOD were detected by the FDA method (the FDA-1 LOD is 0.010 ng/mg and FDA-2 LOD is 0.005 ng/mg) but were reported as not detected by the private laboratory (i.e., Sample #s 21, 26, 29, 31, and 35), although the private laboratory claimed its method was more sensitive than the FDA method (13). To investigate these discrepancies, the FDA laboratory replicated the private laboratory analytical procedure to the extent possible.

### Replication of Private Laboratory LC/MS Method by FDA

The private laboratory LC/MS method was replicated with the reported HPLC conditions and quantitation methods (i.e.*,* stable isotope labeled NDMA was used as an internal standard and a weighted (1/×) calibration curve was constructed for quantitation). Because the QToF instrument used by the private laboratory was not available in the FDA laboratory, for the replication study mass spectrometric detection was performed with an Orbitrap technology platform. A comparison between the mass spectrometric conditions described by the private laboratory and used by the FDA laboratory is listed in Table 2.

**Table 2:** Comparison of mass spectrometry (MS) conditions used in this study (FDA) and the private laboratory method description

MS Conditions	Private laboratory	FDA
Instrument	QToF	Orbitrap
Ionization mode	APCI, positive	APCI, positive
Data acquisition	MRMHR	Targeted MS2
MS scan	50–450 m/z	40–90 m/z
Mass resolution	> 25,000^a^	45,000^b^
Transition(s)	75.0553 → 75.0553	75.0553 → 75.0553
	83.0997 → 83.0997	83.0997 → 83.0997

^ab^The maximum resolution is specified as ≥ 42,000 (FWHM) at *m/z* 956 for this instrument; The maximum resolution is specified as 480,000 at *m/z* 200 for this instrument

The abbreviations “MRMHR” and “Targeted MS2” are the same data acquisition technique (although named differently by the two instrument vendors) through which the protonated NDMA ion and its isotope-labeled equivalent are isolated and undergo fragmentation. The mass-to-charge ratio (m/z) of certain fragment ion(s) or the remaining precursor ions in the collected mass spectra are then used for quantitation through extracted ion chromatograms (EICs). The mass spectra were collected at different mass scan ranges (50–450 m/z for the private laboratory and 40–90 m/z for the FDA study). The mass range does not affect the results, provided that the ions used for quantitation (m/z 75.0553 and 83.0997) are included in the scan ranges. Overall, the mass spectrometric detection approach used by the FDA was consistent with that described in the private laboratory analytical procedure.

The replication of the chromatographic characteristics of the private laboratory method at the FDA was successful. The retention times observed by FDA for NDMA and the stable isotope labeled NDMA peak were 7.39 min and 7.36 min (Fig. S-1, Supplemental Data_1_Figures), consistent with that reported by the private laboratory (7.48 min) (13). The slight shift noted in the elution time was likely due to plumbing differences between the HPLC systems. The spiked standard approach used by the FDA and the stable isotopically enriched NDMA used by the private laboratory produced a linear response across the test range albeit with different units (for FDA concentration of standard versus response ratio, while for the private laboratory concentration ratio to internal standard versus response ratio). Application of the stable isotopically enriched standard approach to measure NDMA amounts in three selected examples (Sample #s 13, 14, and 21 from Table 1) of the private laboratory samples with a mass tolerance of ± 15 ppm yielded the same values observed by the FDA. Thus, one possible explanation for the difference in the response was either insufficient mass accuracy in the performance of the instrument or wide mass tolerance in the analysis of the data by the private laboratory.

### Necessary Mass Accuracy and Mass Tolerance Settings to Distinguish NDMA from DMF

The compound in the metformin drug product sample that may interfere with NDMA quantitation was identified as *N,N*-dimethylformamide (DMF) by matching the accurate mass, fragmentation pattern, and retention time to a DMF reference standard. The product ion mass spectrum of m/z 74 (nominal m/z value of the DMF monoisotopic ion) for the sample demonstrates the same pattern as that of the DMF standard (Fig. S-2, Supplemental Data_1_Figures). Both mass spectra contained the DMF monoisotopic ion at m/z 74.0597 (observed value where the theoretical exact mass is 74.0600) and a *N,N*-dimethylamine fragment ion at m/z 46.0650 with the signal ratio of these two ion peaks matching. The EIC peaks of m/z 74.0597 and 46.0650 show that the DMF reference standard elutes at 7.36 min with the same retention times also observed for the metformin sample (Fig. S-2, Supplemental Data_1_Figures). Importantly, the potential for DMF to interfere with NDMA quantitation only occurs where DMF and NDMA co-elute, as was observed with the LC conditions of the private laboratory method (Fig. S-3, Supplemental Data_1_Figures). Furthermore, DMF in pharmaceuticals is allowed as per the ICH Q3C(R6) guideline to be up to 880 ppm (ng/mg).

The FDA hypothesized that the potential cause of the high values reported by the private laboratory was the presence of an interfering substance (i.e.*,* DMF) which co-eluted with NDMA and that the private laboratory method did not provide the needed selectivity for NDMA in the presence of DMF (due to insufficient mass resolution or accuracy in data acquisition or inappropriate mass tolerance setting in data processing). In the product ion mass spectrum of DMF, the ion at m/z 74.0600 represents the calculated monoisotopic ion of DMF. Two ion peaks in the mass spectra from isotopic ions of DMF are potential interferences with NDMA depending on the resolution as follows: (1) m/z 75.0569 from the replacement of ^14^N by ^15^N and (2) m/z 75.0631 from the replacement of one ^12^C by ^13^C. Because the difference between the DMF isotopic ion at m/z 75.0569 and the NDMA ion is 0.0016 amu or 21 ppm, the DMF ^15^N-isotopic ion can be mistakenly identified as the NDMA ion when an insufficient mass accuracy is applied for data acquisition and/or a wide mass tolerance window is used for data processing. The ^13^C-isotope has a 104 ppm difference from NDMA so is less likely to interfere. Thus, the role of the ^15^N-isotope was examined further as a potential interferent that would result in the overestimation of NDMA or in false positive measurements where no NMDA was present in metformin samples containing relatively high amounts of DMF.

**Fig. 1:**
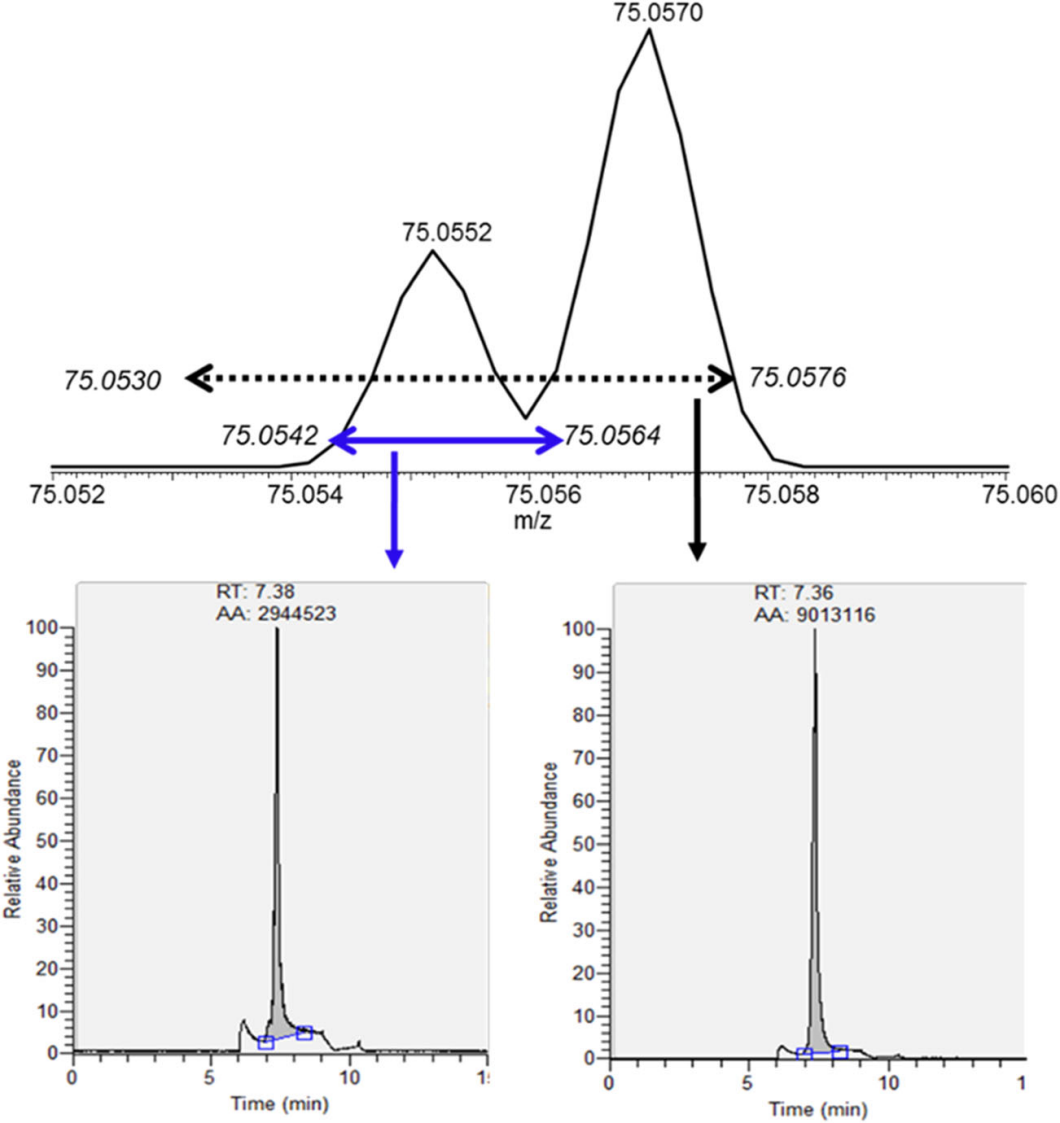
Mass spectra of Sample #13 (ER drug product) spiked with 20 ng/mL of NDMA which also contained DMF (top) and EICs (bottom) demonstrating the overestimation (integrated area of 2,944,523 with ± 15 ppm mass tolerance in the left panel (blue bar), while there is an integrated area of 9,013,116 with ± 30 ppm mass tolerance in the right panel (dotted bar)) of NDMA as the results of DMF interference from C_3_H_7_^15^NO

The risk of interference is illustrated by the mass spectra of the Sample #13 metformin product sample spiked with 20 ng/mL NDMA, in which the protonated NDMA ion (m/z 75.0553) and the DMF isotopic ion (m/z 75.0569) were both present (Fig. 1, top). When the EIC of NDMA was performed at a mass tolerance of ± 15 ppm, any ion peak with m/z values between 75.0542 and 75.0564 would be included as shown by the blue bar in Fig. 1. When a wider mass tolerance window of ± 30 ppm was applied, the m/z range was expanded, and now any ions with m/z values between 75.0530 and 75.0576 would be included (dotted line range) (Fig. 1). Therefore, the EIC peak with mass tolerance window of ± 30 ppm included not only the NDMA ion peak but also the DMF isotopic ion peak, resulting in the EIC peak area increasing by threefold from 2,944,523 (Fig. 1, bottom left) to 9,013,116 (Fig. 1, bottom right).

Alternately, the impact of the instrument mass resolution and mass accuracy on this measurement can be assessed by simulated mass spectra. Xcalibur Qual Browser (Thermo Scientific) was used to simulate spectra at a mass resolution of 25,000 (FWHM) (data not shown). At a resolution of 25,000, NDMA and the DMF ^15^N isotopic ion were observed to merge into one ion peak with an apparent *m/z* value of 75.0567. The mass difference between this simulated unresolved ion peak and the theoretical *m/z* value of NDMA (75.0553) is 19 ppm ((75.0567–75.0553)/75.0553 × 10^6^). Thus, in the simulated data when the mass accuracy of the instrument was insufficient in the data acquisition, the observed *m/z* value of the merged ion peak were shifted, and, as a result, the ion peak would be mistaken as NDMA ion even with a mass tolerance window of ± 15 ppm or narrower applied for EICs in the analysis.

### Comparison of Authentic Samples

The interference of DMF was further demonstrated by the correlation between the DMF levels present in three metformin samples and the apparent NDMA levels reported by the private laboratory. Estimated by the EIC peak areas, the DMF level in Sample #13 was about 10 to 20 times that present in Sample #14 or Sample #21 (Table 3). The DMF level in Sample #21 was the lowest at about half that present in Sample #14. A high level of NDMA was consistently reported for Sample #13 by the private laboratory, while no NDMA was detected by FDA-1 testing (Table 3). NDMA was found in Sample #14 by the private laboratory and FDA-1 testing, but the private laboratory value was about 1.5 times that of FDA’s testing result. NDMA was reported as not detected for Sample #21 by the private laboratory where the FDA-1 observed trace values and consistently, Sample #21 contained the lowest DMF among the three sets of samples.

**Table 3:** DMF levels, reported testing results, and the impact of mass tolerance window on NDMA measurement

Sample # (Table 1)	DMF EIC peak area	Prior results (ng/mg)^a^		Results from FDA replication study (ng/mg)^b^	
		FDA-1	Private laboratory	Mass tolerance ± 15 ppm	Mass tolerance ± 30 ppm
13	4.62E + 09	ND	0.276	ND	0.575
14	3.90E + 08	0.121	0.180	0.131	0.179
21	2.37E + 08	0.010	ND	ND	0.021

^ab”^Prior results” are the values reported by the FDA-1 method or the private laboratory (see Table 1), Results from FDA study are obtained by FDA’s replication of the private laboratory’s method

### Measured NDMA Levels in Metformin Products Are Affected by the Mass Tolerance Used for EICs

As a final examination of the role of mass accuracy and mass tolerance in the discrepancy between the FDA and the private laboratory testing results, a comparison was made between analysis of the same data at different mass tolerance settings. The three drug products in Table 3 were compared where the NDMA results represent the following three cases: (1) NDMA was not detected by the FDA but was found at amounts above AI by the private laboratory (e.g.*,* Sample #13); (2) the FDA and the private laboratory results were consistent in that NDMA was either not detected or at the LOD level (e.g.*,* Sample #21); or (3) the FDA and the private laboratory results were consistent in that the NDMA level exceeded the AI although the private laboratory value was slightly higher than the FDA value (e.g.*,* Sample #14). The samples were prepared for these three drug products by adding an appropriate volume of methanol (which was the same solvent used by the private laboratory) containing 40 ng/mL isotope-labeled NDMA internal standard to the crushed tablets and were analyzed by the private laboratory’s method as adapted by the FDA.

The primary observation from these experiments was the significant impact of the mass accuracy of the mass spectrometer in the data acquisition and mass tolerance window applied in data processing after the experimental data had been collected on the reported NDMA results. In the case of Sample #13, when the mass tolerance applied for data processing was changed from ± 15 to ± 30 ppm, the resultant NDMA value rose from not detected to 0.575 ng/mg (< 10–1150 ng for maximum daily dose of 2000 mg for an ER formulation). The degree of impact on the reported results is correlated with the DMF content of the three samples (See Table 3 above). By contrast, the measured NDMA amount in Sample #21 increased slightly from “not detected” to about 0.021 ng/mg (< 10–42 ng in 2000 mg). Similarly, the NDMA amount in Sample #14 also increased from 0.131 to 0.179 ng/mg (262–358 ng in 2000 mg). Notably, the NDMA amount quantitated at ± 30 ppm mass tolerance was close to the value reported by the private laboratory for Sample #14 but was doubled for Sample #13. The reason for the difference in the Sample #13 value is not clear. Overall, the results generated from the mass tolerance of ± 15 ppm are in good agreement with the FDA testing results, while the results from the ± 30 ppm mass tolerance are more consistent with what the private laboratory reported (Table 3).

### Relative DMF Levels in the Private Laboratory Sample Set

**Fig. 2:**
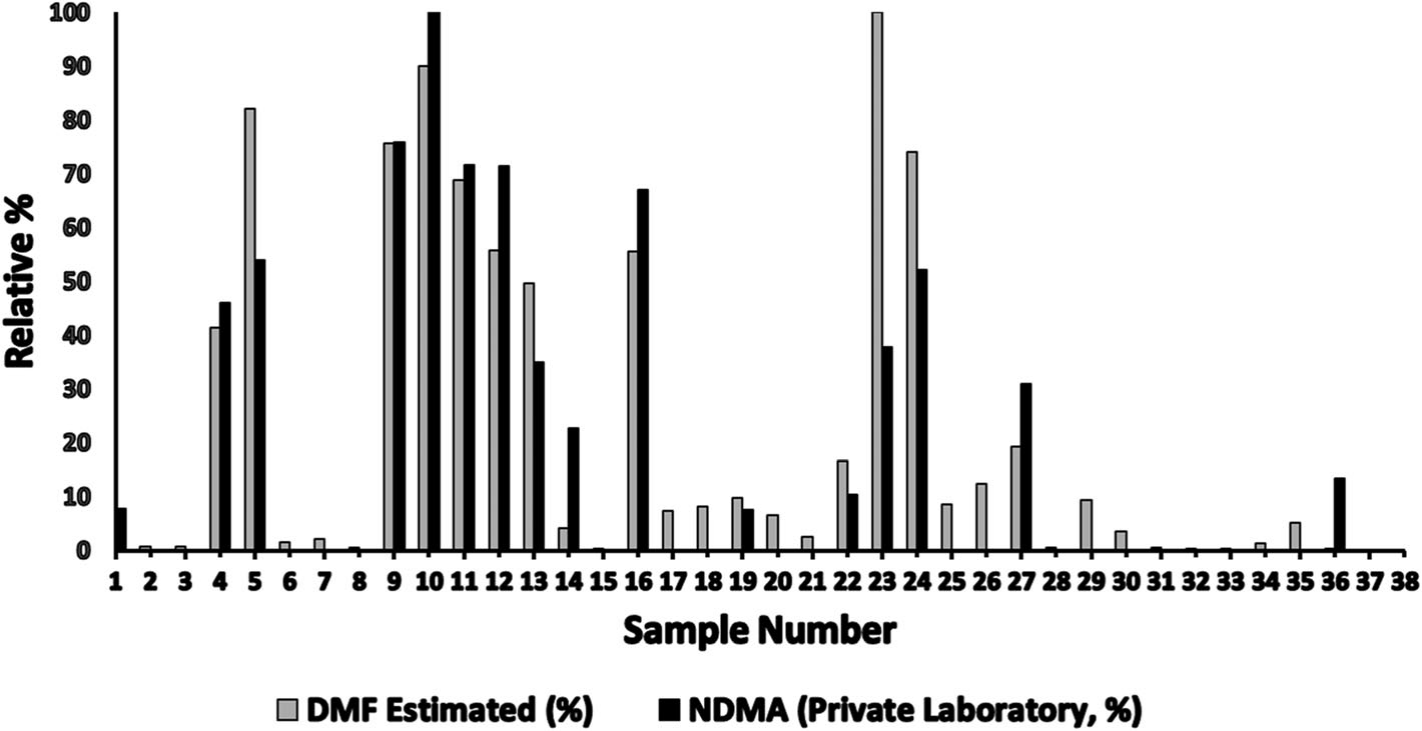
A plot of the relative percentage versus the sample number in the private laboratory samples. The relative percentages are from each value being divided by the highest value reported in the set by the private laboratory for NDMA (or zero where no amount was detected). Similarly, the relative amount of DMF in these samples (from FDA measurements) was plotted for the same samples. The results have a coefficient of determination of 0.81

As shown in Table 3 the amounts of DMF present in the 38 samples can be estimated based on the EICs (using the exact mass of DMF with ± 15 ppm mass tolerance) compared with a 100 ng/mL DMF standard prepared in methanol. Figure 2 shows an overlay of the relative amount of NDMA reported by the private laboratory and the relative amount of DMF (as estimated from the FDA MS data). For comparison purposes, the DMF and NDMA values, separately, were divided by the highest value measured across the 38-sample set for each analyte to generate a percent of the maximum value observed for each sample. The amount of DMF and the amount of NDMA observed at the private laboratory are correlated with a coefficient of determination of 0.81. Similarly, the same comparison can be made with NDMA values reported by FDA across the 38 samples. By contrast to the correlation observed in Fig. 2 for the private laboratory data, the FDA data show a coefficient of determination of 0.26, indicating little to no correlation between DMF amounts present in the samples and the measured amount of NDMA.

## Discussion

The private laboratory reported testing results to the Agency in a Citizen Petition. The FDA responded to the Citizen Petition by testing these products and noted different results (although 8 of the 38 samples from 5 firms did have NDMA amounts above the AI). In this set of 38 samples, only the ER products had detectable NDMA amounts as tested by FDA. The Agency responded by requesting voluntary recalls from the firms manufacturing these products. These firms complied with the request (16). Subsequently, the private laboratory hypothesized that because the FDA had not used a stable isotope-enriched standard for NDMA in the FDA method the agencies results were an underestimation of the NDMA amounts in the US metformin supply. In addition, the private laboratory “crowd sourced” samples from the US market tested them using the same method described above and provided the results in a non-peer reviewed preprint manuscript (15) which reported amounts of NDMA above the AI in 36% of the metformin drugs tested. As described in this study, the use of a stable isotope enriched standard for NDMA as internal control did not prevent the co-elution of DMF from causing overestimation of the NDMA amounts in many products when using the private laboratory approach.

The results of FDA’s analytical procedure validation experiments showed that the FDA tests developed to measure the nitrosamine amounts in metformin drugs had adequate analytical procedure performance. Furthermore as an added check, the FDA developed orthogonal methods that used different ionization sources for the MS detection and different chromatographic conditions (columns/gradients). Comparable results were observed across these measurements on the private laboratory samples. If there were matrix effects with one method, these would be unlikely to be the same with a second method. These findings provide a high degree of confidence in the accuracy of the FDA methods and measurements that have been developed and performed, respectively.

Importantly, the presence of an interfering substance like DMF co-eluting with NDMA needs to be addressed in the analytical procedure in the settings of the mass spectrometer detection parameters and in the analysis of the data. For HRMS-based quantitation, mass resolution and mass accuracy applied in the data acquisition and mass tolerance used in the data processing have an interwoven impact on the outcome as demonstrated in the data reported here (17). The experimental data showed that in the case of co-elution between DMF and NDMA, without a sufficient mass accuracy in the data acquisition and/or sufficient mass tolerance in data analysis, the test signal would be higher than the true value of NDMA because the integrated EIC peak area would be higher by 0.4% of the DMF present in the sample and would yield a false higher signal.

## Conclusions

The results of the study described here indicate that care must be taken to ensure analytical procedure specificity in the presence of potential interfering substances in the development and validation of analytical tests for drugs like metformin. Often, extensive knowledge of the drug substance and drug product properties, manufacturing processes, and the potential impurities that could interfere with an analytical measurement are required to assure sufficient analytical specificity. In this case, an isotopic peak associated with an allowed DMF impurity was shown to impact the private laboratory’s method only when insufficient mass tolerance was used. The data show the value of having the necessary measurement resolution to assure the specificity of an analytical procedure for small molecule analytes such as nitrosamine compounds present in ppm (ng/mg) to ppb (pg/mg) amounts. Furthermore, the data show that orthogonal methods provide assurance that matrix effects are not impacting the measurements and serve to confirm NDMA levels and the accuracy of the reported values.

## Electronic supplementary material

ESM 1 (DOCX 103 kb)

ESM 2 (DOCX 476 kb)

## References

[1] Gushgari AJ, Halden RU (2018). Critical review of major sources of human exposure to N-nitrosamines. Chemosphere..

[2] Kocak D, Ozel MZ, Gogus F, Hamilton JF, Lewis AC (2012). Determination of volatile nitrosamines in grilled lamb and vegetables using comprehensive gas chromatography - nitrogen chemiluminescence detection. Food Chem.

[3] Park JE, Seo JE, Lee JY, Kwon H (2015). Distribution of seven N-nitrosamines in food. Toxicol Res.

[4] Lim DS, Roh TH, Kim MK, Kwon YC, Choi SM, Kwack SJ, Kim KB, Yoon S, Kim HS, Lee BM (2018). Risk assessment of N-nitrosodiethylamine (NDEA) and N-nitrosodiethanolamine (NDELA) in cosmetics. J Toxicol Environ Health A.

[5] Charoo NA, Ali AA, Buha SK, Rahman Z (2019). Lesson learnt from recall of valsartan and other angiotensin II receptor blocker drugs containing NDMA and NDEA impurities. AAPS PharmSciTech.

[6] Parr MK, Joseph JF (2019). NDMA impurity in valsartan and other pharmaceutical products: analytical methods for the determination of N-nitrosamines. J Pharm Biomed Anal.

[7] Shepard EA, Nawarskas JJ. Nitrosamine impurities in angiotensin receptor blockers. Cardiol Rev. 2020. 10.1097/CRD.0000000000000323 32467427

[8] FDA/CDER. FDA updates and press announcements on NDMA in metformin FDA.gov2020 [Available from: https://www.fda.gov/drugs/drug-safety-and-availability/fda-updates-and-press-announcements-ndma-metformin.

[9] EDQM. Ad-hoc projects of the OMCL Network EDQM.EU2020 [Available from: https://www.edqm.eu/en/ad-hoc-projects-omcl-network.

[10] FDA/CDER. FDA updates and press announcements on angiotensin II receptor blocker (ARB) recalls (Valsartan, Losartan, and Irbesartan) 2020 [Available from: https://www.fda.gov/drugs/drug-safety-and-availability/fda-updates-and-press-announcements-angiotensin-ii-receptor-blocker-arb-recalls-valsartan-losartan.

[11] FDA/CDER. FDA updates and press announcements on NDMA in Zantac (ranitidine) 2020 [Available from: https://www.fda.gov/drugs/drug-safety-and-availability/fda-updates-and-press-announcements-ndma-zantac-ranitidine.

[12] CDER/FDA. Laboratory tests | Metformin 2020 [Available from: https://www.fda.gov/drugs/drug-safety-and-availability/laboratory-tests-metformin.

[13] Valisure. Valisure citizen petition on metformin 2020 [Available from: https://www.valisure.com/wp-content/uploads/Valisure-FDA-Citizen-Petition-on-Metformin-v3.9.pdf.

[14] FDA/CDER. FDA alerts patients and health care professionals to nitrosamine impurity findings in certain metformin extended-release products FDA.gov: US FDA; 2020 [Available from: https://www.fda.gov/news-events/press-announcements/fda-alerts-patients-and-health-care-professionals-nitrosamine-impurity-findings-certain-metformin.

[15] Wu Q, Kvitko E, Jessop A, Williams S, Constantino RC, Kucera K, et al. Analysis of crowdsourced metformin tablets from individuals reveals widespread contamination with N-Nitrosodimethylamine (NDMA) in the United States. medRxiv preprint. 2020.

[16] CDER/FDA. Recalls, market withdrawals, & safety alerts FDA.gov: US FDA; 2020 [Available from: https://www.fda.gov/safety/recalls-market-withdrawals-safety-alerts.

[17] Xian F, Hendrickson CL, Marshall AG (2012). High resolution mass spectrometry. Anal Chem.

