# Supplementary material for: A Cautionary Tale: Quantitative LC-HRMS Analytical Procedures for the Analysis of N-Nitrosodimethylamine in Metformin
Source: AAPS J. 2020 Jul 1;22(4):89. doi: 10.1208/s12248-020-00473-w (PMC7329790; doi:10.1208/s12248-020-00473-w)
Supplement: Supplementary file 1 — (DOCX 103 kb) [file 12248_2020_473_MOESM1_ESM.docx]

**Supplemental Data_1: Figures**


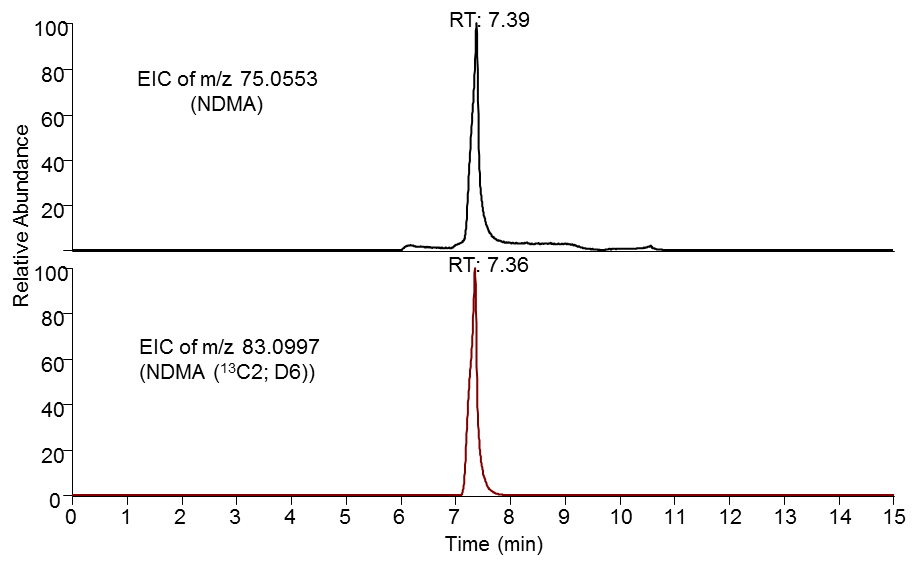


**Figure S-1:** Extracted ion chromatograms (EIC) of NDMA (top) and NDMA (^13^C2; D6) (bottom). NDMA elutes at 7.39 min, and NDMA (^13^C2; D6) elutes at 7.36 min.


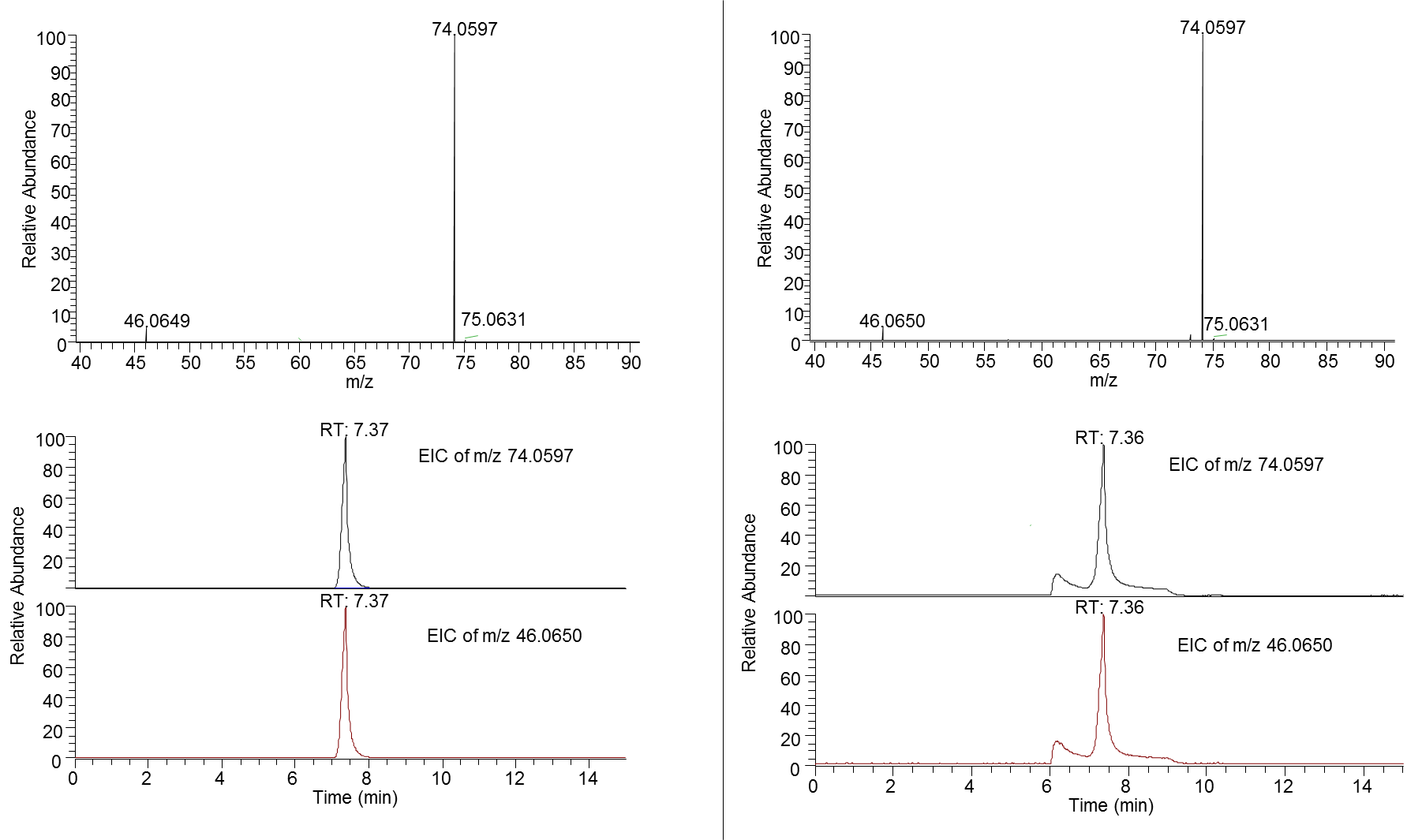


| **Sample** | **DMF standard** |
| --- | --- |

**Figure S-2:** Product ion mass spectra of m/z 74 (top) and the extracted ion chromatograms (EIC) of m/z 74.0597 and 46.0650 (bottom) for the metformin drug product sample (left) the DMF reference standard (right).


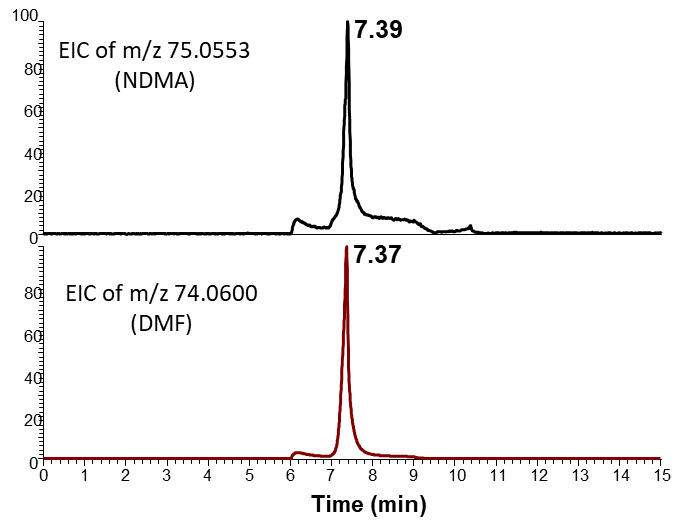


**Figure S-3:** Co-elution of NDMA and DMF in the chromatography used by the private laboratory. The EIC of the exact mass of NDMA (eluting at 7.39 min) and EIC of the exact mass of DMF (eluting at 7.37 min) are indicated in the Figure.
