# Supplementary material for: A Cautionary Tale: Quantitative LC-HRMS Analytical Procedures for the Analysis of N-Nitrosodimethylamine in Metformin
Source: AAPS J. 2020 Jul 1;22(4):89. doi: 10.1208/s12248-020-00473-w (PMC7329790; doi:10.1208/s12248-020-00473-w)

**Supplemental Data_2_Methods**

**Supplemental Table S-1** Liquid Chromatography conditions for FDA-1 and FDA-2 methods

|  | FDA-1 | | FDA-2 | |
| --- | --- | --- | --- | --- |
| HPLC Column | XSelect CSH C18 2.5 µm, 3.0 x 150 mm (Waters, Part No. 186006728) | | Kinetex BiPhenyl, 150 x 3.0 mm, 2.6 µm (Phenomenex; P/N 00F-4622-Y0) | |
| Column Temp. | 30 °C | | 40 °C | |
| Flow Rate | 0.3 mL/min | | 0.4 mL/min | |
| Gradient: (A: 0.1% formica acid in water; B: 0.1% formic acid in methanol) | Time (min) | % B | Time (min) | % B |
|  | 0 | 10 | 0 | 5 |
|  | 5 | 10 | 3 | 5 |
|  | 6 | 90 | 5 | 10 |
|  | 9 | 90 | 6 | 60 |
|  | 9.1 | 10 | 10 | 60 |
|  | 14 | 10 | 13 | 80 |
|  | - | - | 13.1 | 100 |
|  | - | - | 15 | 100 |
|  | - | - | 15.1 | 5 |
|  | - | - | 18 | 5 |
| Injection Volume | 3 µL | | | |

**FDA-1-Liquid Chromatography-High Resolution Mass Spectrometry (LC-HRMS) Method for the Determination of NDMA in Metformin Drug Substance and Drug Product**

**Background:** Metformin is a popular prescription medication for the treatment of type 2 diabetes. It was suspected being contaminated with N-nitroso-*di*-methylamine (NDMA), a probable human carcinogen, according to the testing results reported by Valisure, an online pharmacy claiming to re-test all the medicines it sells. Correspondingly, OTR developed a liquid chromatography-high resolution mass spectrometry (LC-HRMS) method to determine the level of NDMA in metformin drug products and drug substances to assist the on-going investigations.

**Summary:**

A LC-HRMS method was developed and validated following ICH Q2 (R1) for the detection and quantitation of NDMA in metformin drugs. The limit of detection (LOD), limit of quantitation (LOQ) and range of the method are summarized below:

|  | **NDMA** |
| --- | --- |
| LOD (ng/mL) | 1.0 |
| (ppm) | 0.01 |
| LOQ (ng/mL) | 3.0 |
| (ppm) | 0.03 |
| Range (ng/mL) | 3.0 - 10 |
| (ppm) | 0.03 – 0.1 |

**Purpose**

This method is used to quantitate N-nitroso-*di*-methylamine (NDMA) impurity in metformin drug substance or drug product.

**Principle**

N-nitroso-*di*-methylamine (NDMA) impurity is separated from metformin by reverse phase chromatography and is detected by a high-resolution and high-mass accuracy (HRAM) mass spectrometer. High sensitivity detection is achieved by monitoring the accurate *m/z* value of the protonated NDMA ion. Quantitation is performed by comparing the peak area of the NDMA impurity in extracted ion chromatogram of the samples to the peak area of the NDMA reference standard in external calibration standard.

**Reagents**

- NDMA Reference Standard
- Formic acid, LC/MS grade (Fisher A117-50 or equivalent)
- Methanol, LC/MS grade (Fisher A456-4 or equivalent)
- Water, LC/MS grade or equivalent

**Equipment/Instrument**

- HPLC or UHPLC system equipped with temperature-controlled autosampler and column compartment
- Q Exactive^TM^ hybrid quadrupole-orbitrap mass spectrometer (Thermo-Fisher Scientific)
- HPLC column: XSelect CSH C18 2.5 µm, 3.0 x 150 mm, P/N 186006728
- Analytical Balance
- Vortex Mixer
- 15 mL glass centrifuge tubes
- Wrist action shaker
- 0.22 µm PVDF syringe filters
- Centrifuge
- HPLC vials

**Mobile phase preparation**

- Mobile phase A (0.1% formic acid in water): mix formic acid and water at a volume ratio of 1:1000
- Mobile phase B (0.1% formic acid in methanol): mix formic acid and methanol at a volume ratio of 1:1000

**Diluent and Blank**: Methanol

**NDMA Intermediate Stock Standard preparation (100 ng/mL)**

Prepare a 100 ng/mL intermediate stock standard solution in methanol using commercially available NDMA reference stock standard solution.

**Working Standard Preparation (3.0 ng/mL)**

Transfer a 3.0 mL aliquot volume of the intermediate stock standard into a 100 mL volumetric flask and dilute to volume with methanol. Prepare fresh daily.

**Drug substance sample preparation**

Accurately weigh 500 mg of drug substance into a 15 mL glass centrifuge tube. Add 5.0 mL of methanol and mix the solution using a vortex mixer. Shake the sample for 40 minutes using a mechanical wrist action shaker.

**Drug product sample preparation**

Crush the appropriate number of tablet(s) to obtain a target concentration of 100 mg/mL of API in methanol, and transfer into a 15 mL glass centrifuge tube. Add methanol and mix for about a minute using a vortex mixer. Shake the sample for 40 minutes using a mechanical wrist action shaker.

After extraction, centrifuge the sample for 15 minutes at 4500 rpm. Filter the supernate using a 0.22 µm PVDF syringe filter, discard the first 1 mL and transfer the filtered sample into an HPLC vial for analysis.

**Chromatographic Conditions**

| **HPLC Column** | XSelect CSH C18 2.5 µm, 3.0 x 150 mm (Waters, Part No. 186006728) | | |
| --- | --- | --- | --- |
| **Column Temp.** | 30 °C | | |
| **Flow Rate** | 0.3 mL/min | | |
| **Mobile Phase A** | 0.1% formic acid in water | | |
| **Mobile Phase B** | 0.1% formic acid in methanol | | |
| **Gradient** | Time (min) | A% | B% |
|  | 0 | 90 | 10 |
|  | 5.0 | 90 | 10 |
|  | 6.0 | 10 | 90 |
|  | 9.0 | 10 | 90 |
|  | 9.1 | 90 | 10 |
|  | 14.0 | 90 | 10 |
| **Injection Volume** | 3 µL | | |
| **Autosampler Temp.** | 20 °C | | |
| **Needle Wash** | 80:20, Methanol:Water with 0.1% Formic Acid | | |

**Mass spectrometer conditions**

- Instrument

Q Exactive^TM^ mass spectrometer (Thermo-Fisher)

- Ion Source Settings

Note: Ion source parameters can be adjusted to achieve the desired sensitivity.

| **Sheath Gas Flow Rate** | 50 arbitrary units |
| --- | --- |
| **Aux Gas Flow Rate** | 15 arbitrary units |
| **Sweep Gas Flow Rate** | 0 units |
| **Spray Voltage** | 3.5 kV |
| **Capillary Temp.** | 350 °C |
| **Aux Gas Heater Temp.** | 350 °C |

- Scan Settings

Note: 1) The scan start-end time should be adjusted for the user’s HPLC system since the retention time of the NDMA impurity may vary between different HPLC systems, 2) The divert valve can be used to divert the eluent to waste when a scan is not performed.

| **NDMA Impurity** | |
| --- | --- |
| **Scan Type** | PRM |
| **Polarity** | Positive |
| **Scan Start -End (min)** | 3.2 – 5.0 |
| **m/z Isolated for PRM** | 75.0553 |
| **NCE** | 80 |
| **Isolation Window** | 1.5 m/z |
| **Microscans** | 3 |
| **Resolution** | 35,000 |
| **AGC target** | 2e5 |
| **Maximum IT** | 100 ms |

**Injection Sequence**

- Inject Blank (use diluent) at least once at the beginning of a sequence
- Inject working standard solution for six consecutive times before the injection of the first sample
- Inject working standard solution once every six injections of samples and at the end of a sequence.
- Example:

| **Order** | **Solution** | **No. of Injections** |
| --- | --- | --- |
| 1 | Blank | 1 |
| 2 | Standard (3 ng/mL) | 6 |
| 3 | Blank | 1 |
| 4 | Sample 1 | 1 |
| 5 | Sample 2 | 1 |
| 6 | Sample 3 | 1 |
| 7 | Sample 4 | 1 |
| 8 | Sample 5 | 1 |
| 9 | Sample 6 | 1 |
| 10 | Standard (3 ng/mL) | 1 |
| … | … | … |

**System Suitability**

- The % RSD (n = 6) of the NDMA peak areas for the first six injections of the standard solution (3 ng/mL) should be no more than 10%.
- The cumulative % RSD of the NDMA peak areas for working standard should be no more than 15%. (cumulative % RSD of the peak area is calculated by combining the initial six replicate injections of the standard solution and each subsequent bracketing standard).

**Data Processing**

- NDMA peak areas from the extracted ion chromatograms (EIC) with a *m/z* tolerance of 15 ppm are used for quantitation. The NDMA *m/z* value to be extracted is listed below:

| **NDMA** | |
| --- | --- |
| m/z to be extracted | 75.0553 |

- The retention time difference of the NDMA impurity in the analyzed samples should not be more than 2% of the retention time of the corresponding NDMA peak in the reference standard solution.

**Calculation**

***Drug Substance:***

NDMA impurity (ng/mg or ppm) = $\text{A}\text{spl}\text{ }\text{× }\frac{\text{C}\text{s}}{\text{As}}\text{ ×}\text{ }\frac{V}{W}$

| Where: | A_spl_ = Area of the NDMA peak in the sample solution |
| --- | --- |
|  | As = Average area of the NDMA peak in the Working Standard Solution from the first six consecutive injections |
|  | C_s_ = Concentration of the NDMA in Working Standard Solution (ng/mL) |
|  | W = Weight of drug substance (mg) |
|  | V = Volume of the diluent in the sample solution (mL) |

***Drug Product:***

NDMA impurity (ng/mg or ppm) = $\text{A}\text{spl}\text{ }\text{× }\frac{\text{C}\text{s}}{\text{As}}\text{ × }\frac{1}{100 mg/mL}$

| Where: | A_spl_ = Area of the NDMA peak in the sample solution |
| --- | --- |
|  | As = Average area of the NDMA peak in the Working Standard Solution from the first six consecutive injections |
|  | C_s_ = Concentration of the NDMA in Working Standard Solution (ng/mL) |

**Report**

- Report the nitrosamine impurity content in ppm with three significant figures if the value is ≥ LOD
- Report ‘not detected’ if no nitrosamine impurity is detected or the value is < LOD

**Example Chromatograms**

**Methanol Blank**

**
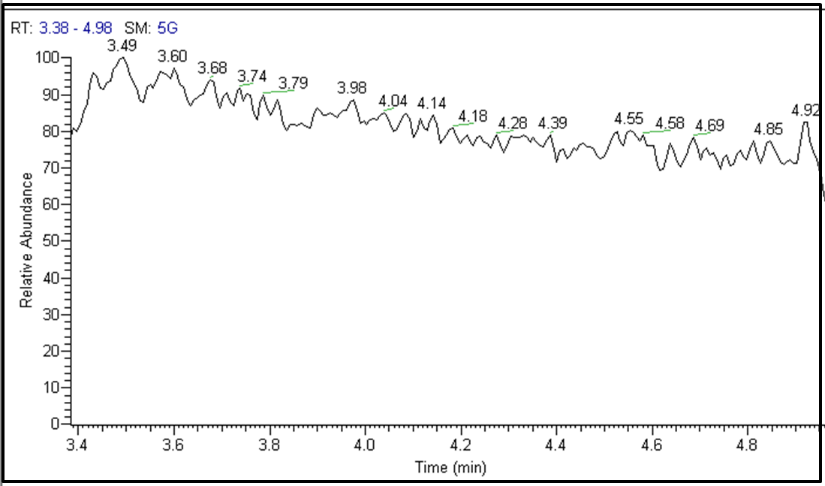
**

**NDMA (3.0 ng/mL Standard)**

**
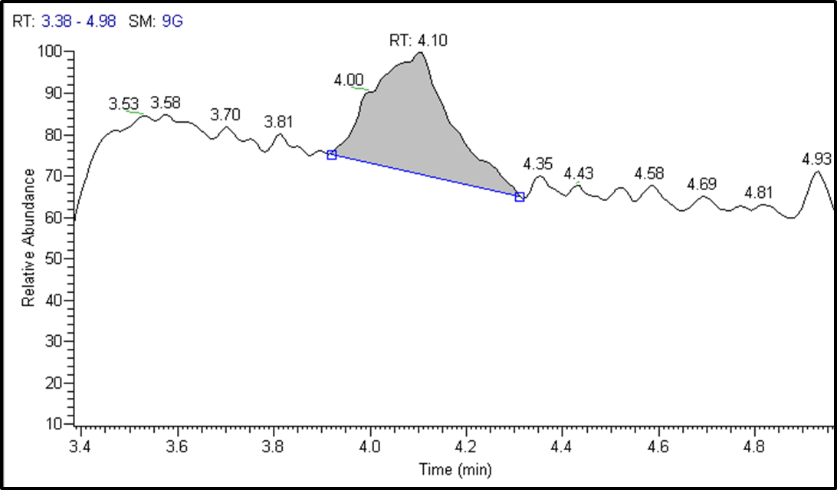
**

**Metformin drug product**

**
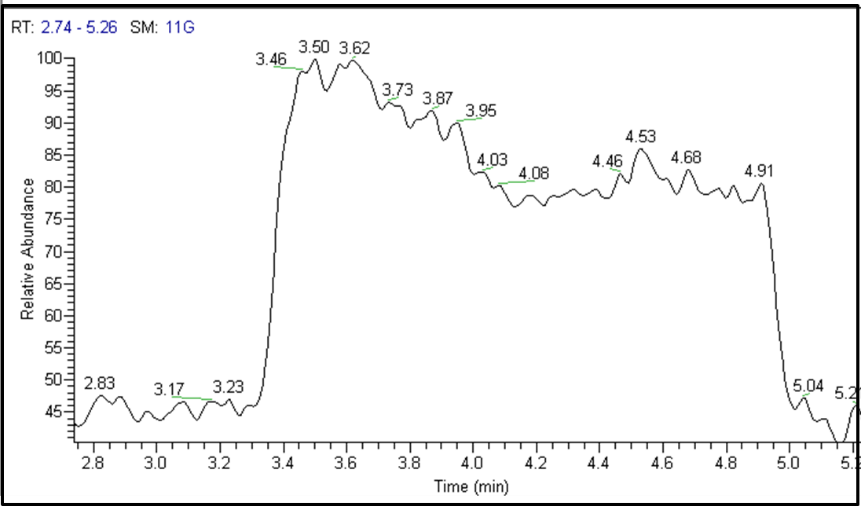
**

**Metformin drug product Spiked with NDMA standard**


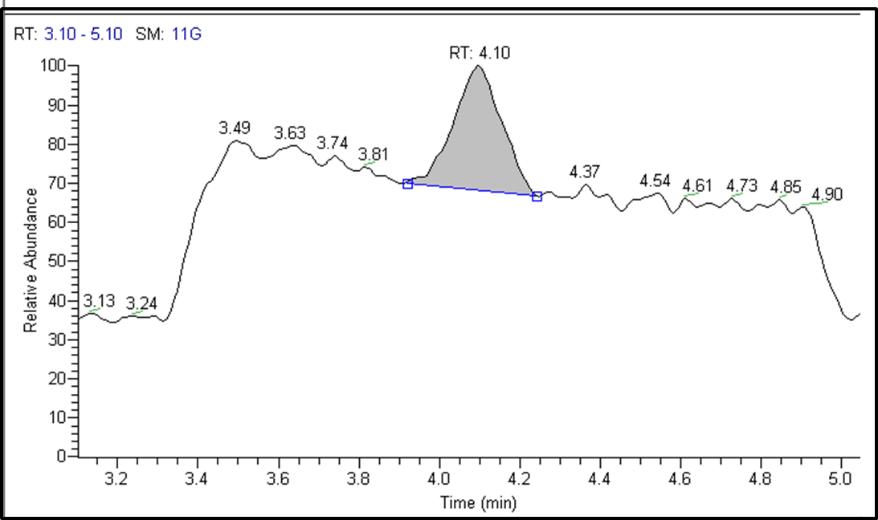


**FDA-2-** **Liquid Chromatography-High Resolution Mass Spectrometry (LC- ESI-HRMS) Method for the Determination of Nitrosamine Impurities in Metformin Drug Substance and Product**

**Background:** Metformin is a prescription drug used to control high blood sugar in patients with type 2 diabetes. NDMA (N-nitroso-dimethylamine) has been classified as a Group 2A compound thereby defining it as “probably carcinogenic to humans.” FDA has set strict daily acceptable intake limits on NDMA in pharmaceuticals of 96 nanograms daily (IR dose is 0.038ppm based on 2550 mg MDD; ER dose is 0.048 ppm based on 2000 mg MDD).

A primary LC-HRMS screen—called FDA-1—above for metformin is in place. Positive NDMA results can be confirmed with this orthogonal method—FDA—2.

**Summary:**

An LC-ESI-HRMS method was developed and validated following ICH Q2(R1) for the detection and quantitation of eight nitrosamine impurities, including N-nitroso-dimethylamine (NDMA), N-nitroso-diethylamine (NDEA), N-ethyl-N-nitroso-2-propanamine (NEIPA), N-nitroso-*di*isopropylamine (NDIPA), N-nitroso-*di*-n-propylamine (NDPA), N-nitroso-methylphenylamine (NMPA), N-nitroso-*di*-n-butylamine (NDBA) and N-nitroso-N-methyl-4-aminobutyric acid (NMBA) in metformin drug substance and drug product. The limit of detection (LOD), limit of quantitation (LOQ) and range of the method are summarized below:

|  | **NDMA** | **NDEA** | **NEIPA** | **NDIPA** | **NDPA** | **NMPA** | **NDBA** | **NMBA** |
| --- | --- | --- | --- | --- | --- | --- | --- | --- |
| LOD (ng/mL) | 0.5 | 0.2 | 0.3 | 0.1 | 0.1 | 0.1 | 0.1 | 0.2 |
| (ppm) | 0.005 | 0.002 | 0.003 | 0.001 | 0.001 | 0.001 | 0.001 | 0.002 |
| LOQ (ng/mL) | 1.0 | 2.0 | 2.0 | 2.0 | 0.5 | 0.5 | 0.5 | 0.5 |
| (ppm) | 0.01 | 0.02 | 0.02 | 0.02 | 0.005 | 0.005 | 0.005 | 0.005 |
| Range (ng/mL) | 1.0 – 10 | 2.0 – 10 | 2.0 – 10 | 2.0 – 10 | 0.5 – 10 | 0.5 – 10 | 0.5 – 10 | 0.5 – 10 |
| (ppm) | 0.01 – 0.1 | 0.02 – 0.1 | 0.02 – 0.1 | 0.02 – 0.1 | 0.005 – 0.1 | 0.005 – 0.1 | 0.005 – 0.1 | 0.005 – 0.1 |

**Purpose**

Development of an orthogonal LC-HRMS method to quantitate the following eight nitrosamine impurities in metformin drug substance or drug product: N-nitroso-dimethylamine (NDMA), N-nitroso-diethylamine (NDEA), N-ethyl-N-nitroso-2-propanamine (NEIPA), N-nitroso-*di*isopropylamine (NDIPA), N-nitroso-*di*-n-propylamine (NDPA), N-nitroso-methylphenylamine (NMPA), N-nitroso-*di*-n-butylamine (NDBA) and N-nitroso-N-methyl-4-aminobutyric acid (NMBA).

**Principle**

The eight nitrosamine impurities (NDMA, NDEA, NEIPA, NDIPA, NDPA, NMPA, NDBA and NMBA) are separated from each other and from metformin by reverse phase chromatography and are detected by a high-resolution and high-mass accuracy (HRAM) mass spectrometer. A high sensitivity of detection is achieved by monitoring the accurate *m/z* values of the protonated or deprotonated impurity ions or their fragments. Quantitation is performed by comparing the peak area of an impurity in extracted ion chromatograms of samples to its standard in an external calibration standard solution containing the reference standards for the eight impurities.

**Reagent**

- Reference standards for NDMA, NDEA, NEIPA, NDIPA, NDPA, NMPA, NDBA and NMBA
- Formic acid, LC/MS grade (Fisher A117-50 or equivalent)
- Methanol, LC/MS grade (Fisher A456-4 or equivalent)
- Water, LC/MS grade or equivalent

**Equipment/Instrument**

- HPLC or UHPLC system equipped with temperature-controlled autosampler and column compartment
- Q Exactive^TM^ hybrid quadrupole-orbitrap mass spectrometer (ThermoFisher Scientific)
- HPLC column: Phenomenex Kinetex^®^ 2.6 µm Biphenyl 100 Å, 150 x 3.0 mm (Part No. 00F-4622-Y0)
- Analytical Balance
- Vortex Mixer
- 15 mL glass centrifuge tubes
- Wrist action shaker
- 0.22 µm PVDF syringe filters
- Centrifuge
- HPLC vials

**Mobile phase preparation**

- Mobile phase A (0.1% formic acid in water): mix formic acid and water at a volume ratio of 1:1000
- Mobile phase B (0.1% formic acid in methanol): mix formic acid and methanol at a volume ratio of 1:1000

**Diluent and Blank**: Methanol

**Mixed Stock Standard preparation**

Prepare a mixed stock standard solution in methanol with the following concentrations.

| **Nitrosamine** | **Conc. (ng/mL)** |
| --- | --- |
| NDMA | 100 |
| NDEA | 100 |
| NEIPA | 100 |
| NDIPA | 100 |
| NDPA | 100 |
| NMPA | 100 |
| NDBA | 100 |
| NMBA | 100 |

**Standard Preparation (3.0 ng/mL)**

Transfer a 0.75 mL aliquot volume of the mixed stock standard into a 25 mL volumetric flask and dilute to volume with methanol. Prepare fresh daily.

**Drug substance sample preparation**

Accurately weigh 400 mg of drug substance into a 15 mL glass centrifuge tube. Add 4.0 mL of methanol and mix the solution using a vortex mixer. Shake the sample for 40 minutes using a mechanical wrist action shaker.

After extraction, centrifuge the sample for 15 minutes at 4500 rpm. Filter the supernatant using a 0.22 µm PVDF syringe filter, discard the first 1 mL and transfer the filtered sample into an hplc vial for LC/MS analysis.

**Drug product sample preparation**

Crush the appropriate number of tablet(s) to obtain a target concentration of 100 mg/mL of API in methanol, and transfer into a 15 mL glass centrifuge tube. Add the appropriate volume of methanol and mix for about a minute using a vortex mixer. Shake the sample for 40 minutes using a mechanical wrist action shaker.

After extraction, centrifuge the sample for 15 minutes at 4500 rpm. Filter the supernate using a 0.22 µm PVDF syringe filter, discard the first 1 mL and transfer the filtered sample into an hplc vial for LC/MS analysis.

**Chromatographic Conditions**

| **HPLC Column** | Phenomenex Kinetex^®^ 2.6 µm Biphenyl 100 Å, 150 x 3.0 mm (Part No. 00F-4622-Y0) | | |
| --- | --- | --- | --- |
| **Column Temp.** | 40 °C | | |
| **Flow Rate** | 0.4 mL/min | | |
| **Mobile Phase A** | 0.1% formic acid in water | | |
| **Mobile Phase B** | 0.1% formic acid in methanol | | |
| **Gradient** | Time (min) | A% | B% |
|  | 0 | 95 | 5 |
|  | 3.0 | 95 | 5 |
|  | 5.0 | 90 | 10 |
|  | 6.0 | 40 | 60 |
|  | 10.0 | 40 | 60 |
|  | 13.0 | 20 | 80 |
|  | 13.1 | 0 | 100 |
|  | 15.0 | 0 | 100 |
|  | 15.1 | 95 | 5 |
|  | 18.0 | 95 | 5 |
| **Injection Volume** | 3 µL | | |
| **Autosampler Temp.** | 21 °C (Room Temperature) | | |
| **Needle Wash** | 80:20, Methanol:Water with 0.1% Formic Acid | | |

**Mass spectrometer conditions**

- Instrument

Q Exactive^TM^ mass spectrometer (ThermoFisher) or Q Exactive^TM^ HF-X mass spectrometer (ThermoFisher)

- ESI Source Settings (apply to both negative and positive modes)

| **ESI Source** |  |
| --- | --- |
| **Sheath Gas Flow Rate** | 55 arbitrary units |
| **Aux Gas Flow Rate** | 15 arbitrary units |
| **Sweep Gas Flow Rate** | 0 units |
| **Spray Voltage** | 3.5 kV |
| **Capillary Temp.** | 400 °C |
| **S-Lens** | 55 (applied to Q Exactive^TM^) |
| **Aux Gas Heater Temp.** | 350 °C |

- Scan Settings

Note:

1) The scan start–end time should be adjusted for the user’s HPLC system since the retention times of the impurities may vary between different HPLC systems

2) The divert valve can be used to divert the eluent to waste when a scan is not performed.

| **Impurity** | **NDMA** | **NMBA** | **NDEA** | **NEIPA** | **NDIPA** | **NDPA** | **NMPA** | **NDBA** |
| --- | --- | --- | --- | --- | --- | --- | --- | --- |
| **Scan Type** | PRM | SIM | SIM | PRM | SIM | SIM | SIM | PRM |
| **Polarity** | Positive | Negative | Positive | Positive | Positive | Positive | Positive | Positive |
| **Scan Start -End (min)** | 3.0 – 6.0 | 7.5 – 8.5 | 8.5 – 9.3 | 9.0 – 10.0 | 9.9 – 11.2 | 9.9 – 11.2 | 11.0 – 12.0 | 13.5 – 14.5 |
| **m/z Isolated for PRM** | 75.0553 | N/A | N/A | 117.1022 | N/A | N/A | N/A | 159.1492 |
| **(N) CE** | 80 | N/A | N/A | 10 | N/A | N/A | N/A | 50 |
| **Isolation Window** | 1.5 m/z | 1.5 m/z | 1.5 m/z | 1.5 m/z | 1.5 m/z | 1.5 m/z | 1.5 m/z | 1.5 m/z |
| **Microscans** | 3 | 3 | 3 | 3 | 3 | 3 | 3 | 3 |
| **Resolution** | 35,000 | 70,000 | 70,000 | 35,000 | 70,000 | 70,000 | 70,000 | 35,000 |
| **AGC target** | 2e5 | 1e6 | 1e6 | 2e5 | 1e6 | 1e6 | 1e6 | 2e5 |
| **Max. IT** | 100 ms | 100 ms | 100 ms | 100 ms | 100 ms | 100 ms | 100 ms | 100 ms |

**Injection Order**

- Inject Blank (use diluent) at least once at the beginning of a sequence
- Inject Standard solution for six consecutive times before the injection of the first sample
- Inject Standard solution once every six injections of samples and at the end of a sequence.
- Example:

| **Order** | **Solution** | **No. of Injections** |
| --- | --- | --- |
| 1 | Blank | 2 |
| 2 | Standard | 6 |
| 3 | Blank | 1 |
| 4 | Sample 1 | 1 |
| 5 | Sample 2 | 1 |
| 6 | Sample 3 | 1 |
| 7 | Sample 4 | 1 |
| 8 | Sample 5 | 1 |
| 9 | Sample 6 | 1 |
| 10 | Standard | 1 |
| … | … | … |

**System Suitability**

- The % RSD of the peak area for each nitrosamine impurity for the first six injections of standard solution should be no more than 10%.
- The cumulative % RSD of the peak area for each nitrosamine impurity should be no more than 15%. (cumulative % RSD of the peak area is calculated by combining the initial six replicate injections of the standard solution and each subsequent bracketing standard)

**Data Processing**

- Peak areas in the extracted ion chromatograms (EIC) with a *m/z* tolerance of 15 ppm are used for quantitation. The *m/z* values to be extracted are listed below:

| Impurity | **NDMA** | **NMBA** | **NDEA** | **NEIPA** | **NDIPA** | **NDPA** | **NMPA** | **NDBA** |
| --- | --- | --- | --- | --- | --- | --- | --- | --- |
| **m/z to be extracted** | 75.0553 | 145.0619 | 103.0866 | 75.0553 | 131.1179 | 131.1179 | 137.0709 | 57.0704, 103.0872, 159.1492 |
| **Ret. Time (min)** | 4.34 | 8.01 | 8.79 | 9.46 | 10.39 | 10.82 | 11.39 | 14.17 |

- The retention time difference of any impurity in the analyzed samples should not be more than 2% of the retention time of the corresponding standard in the standard solution.

**Calculation**

***Drug Substance:***

Nitrosamine impurity (ppm) = $\frac{\text{A}\text{spl}}{\text{As}}\text{× C}\text{s}\text{ ×}\text{ }\frac{1 mg}{1\times{10}^{6}ng}\text{×}\text{ }\frac{V}{W}\times{10}^{6}$

| Where: | Nitrosamine impurity refers to NDMA, NDEA, NEIPA, NDIPA, NDPA, NMPA, NDBA or NMBA  A_spl_ = Area of the nitrosamine impurity peak in the sample solution |
| --- | --- |
|  | As = Average area (n = 6) of the nitrosamine impurity peak from the first six consecutive injections of the standard solution |
|  | C_s_ = Concentration of the nitrosamine impurity in the standard solution (3.0 ng/mL) |
|  | W = Weight of drug substance (mg) |
|  | V = Volume of the diluent in the sample solution (mL) |

***Drug Product:***

Nitrosamine impurity (ppm) = $\frac{\text{A}\text{spl}}{\text{As}}\text{× C}\text{s}\text{ × }\frac{1 mg}{1\times{10}^{6} ng}\text{ × }{\frac{1}{100 mg/mL}\times10}^{6}$

| Where: | Nitrosamine impurity refers to NDMA, NDEA, NEIPA, NDIPA, NDPA, NMPA, NDBA or NMBA  A_spl_ = Area of the nitrosamine impurity peak in the sample solution |
| --- | --- |
|  | As = Average area (n = 6) of the nitrosamine impurity peak from the first six consecutive injections of the standard solution |
|  | C_s_ = Concentration of the nitrosamine impurity in the standard solution (3.0 ng/mL) |

**Report**

- Report the nitrosamine impurity content in ppm with three significant figures if the value is ≥ LOD
- Report ‘not detected’ if no nitrosamine impurity is detected or the value is < LOD

**Reference**

1. *FY20-058-DPA-S:* Liquid Chromatography-High Resolution Mass Spectrometry (LC-HRMS) Method for the Determination of NDMA Metformin Drug Substance and Drug Product.

**Example Chromatograms**

**NDMA (3.0 ng/mL Standard)**

Extracted ion chromatogram of m/z 75.0553 from PRM scan of m/z 75.0553 at a mass accuracy of 15 ppm


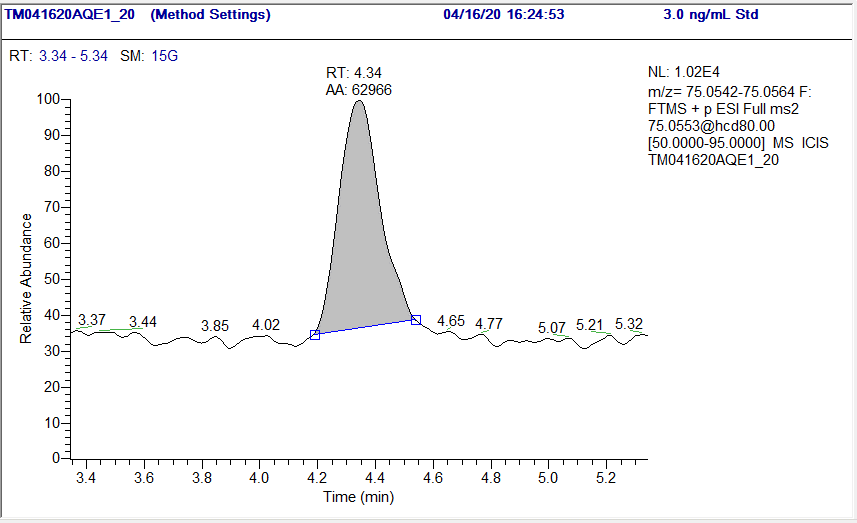


**NMBA (3.0 ng/mL Standard)**

Extracted ion chromatogram of m/z 145.0619 from Targeted-SIM scan of m/z 144.3 to 145.8 at a mass accuracy of 15 ppm


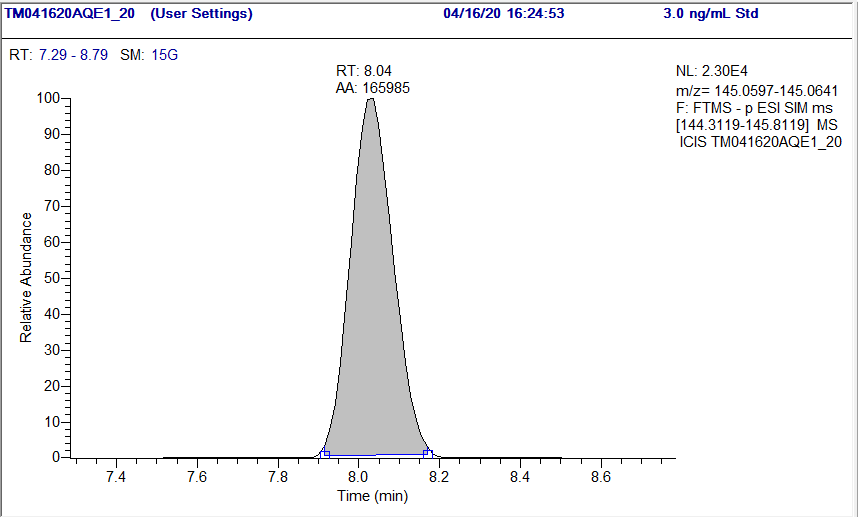


**NDEA (3.0 ng/mL Standard)**

Extracted ion chromatogram of m/z 103.0866 from Targeted-SIM scan of m/z 103.0866 at a mass accuracy of 15 ppm


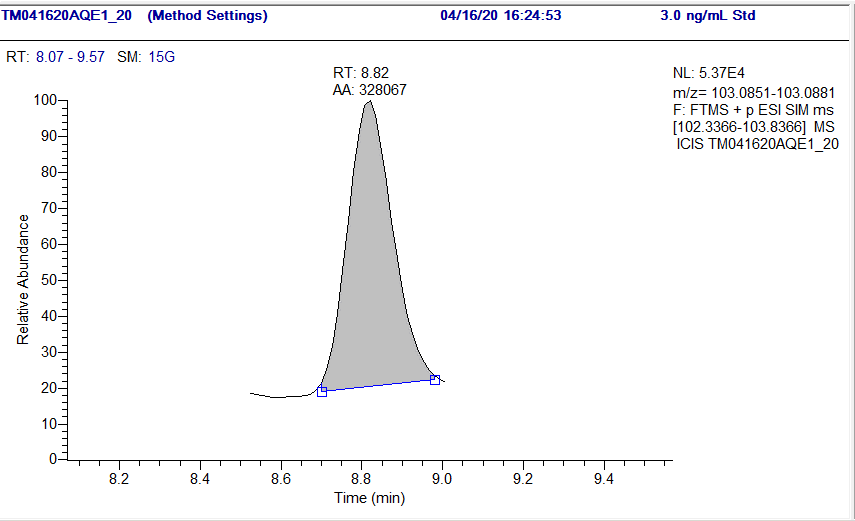


**NEIPA (3.0 ng/mL Standard)**

Extracted ion chromatogram of m/z 75.0553 from PRM scan of m/z 117.1022 at a mass accuracy of 15 ppm


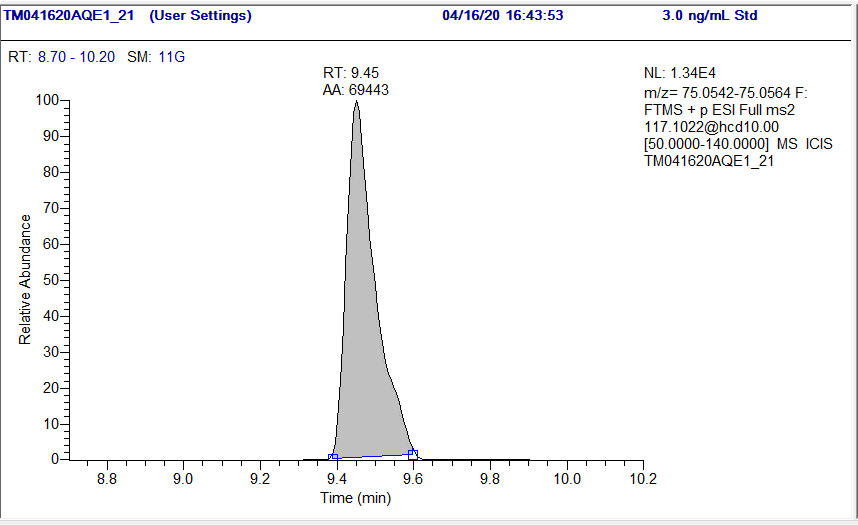


**NDIPA (3.0 ng/mL Standard)**

Extracted ion chromatogram of m/z 131.1179 from Targeted-SIM scan of m/z 130.4 - 131.9 at a mass accuracy of 15 ppm


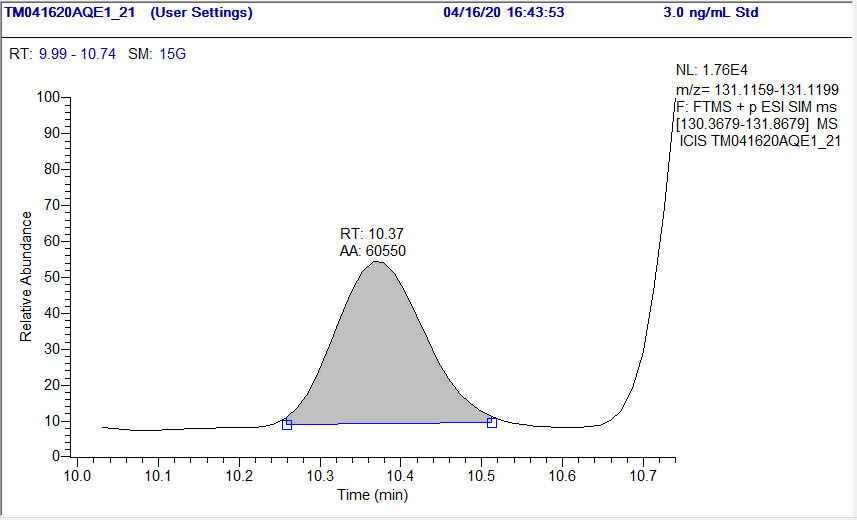


**NDPA (3.0 ng/mL Standard)**

Extracted ion chromatogram of m/z 131.1179 from Targeted-SIM scan of m/z 130.4 - 131.9 at a mass accuracy of 15 ppm


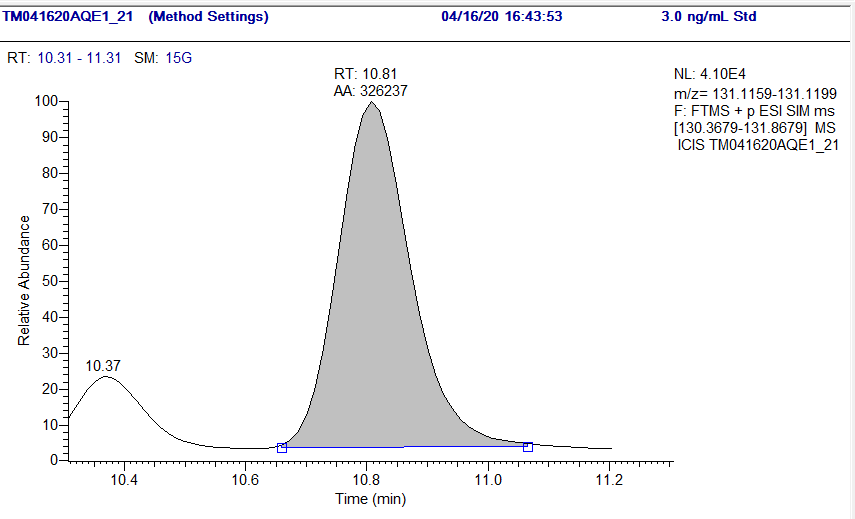


**NMPA (3.0 ng/mL Standard)**

Extracted ion chromatogram of m/z 137.0709 from Targeted-SIM scan of m/z 136.3 - 137.8 at a mass accuracy of 15 ppm


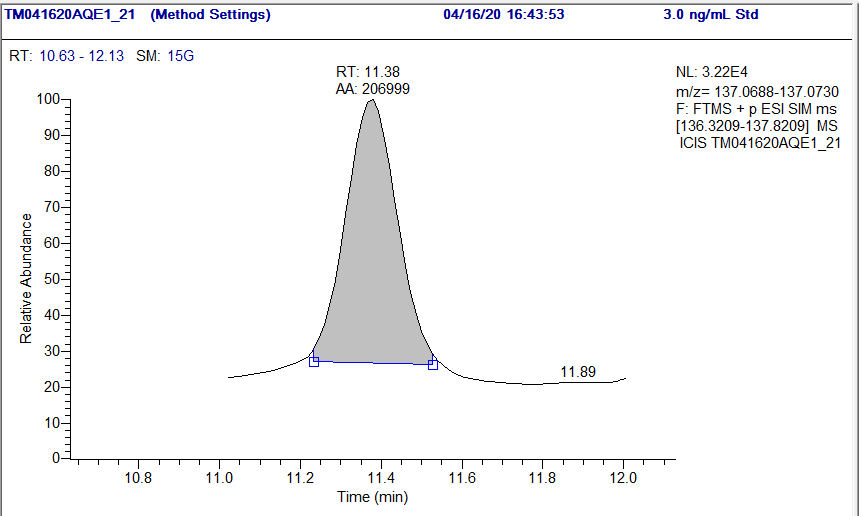


**NDBA (3.0 ng/mL Standard)**

Extracted ion chromatogram of m/z 57.0704, 103.0872 and 159.1492 from PRM scan of m/z 159.1492 at a mass accuracy of 15 ppm


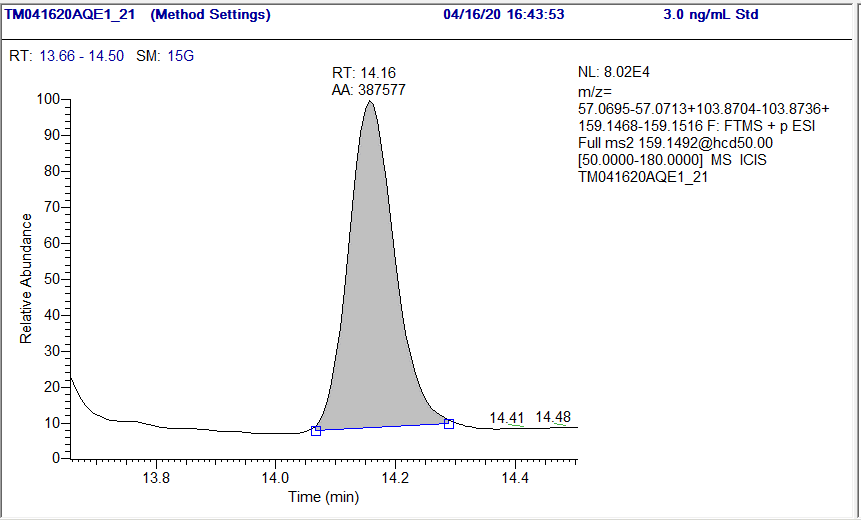

Supplement: Supplementary file 2 — (DOCX 476 kb) [file 12248_2020_473_MOESM2_ESM.docx]
